# Temporal trends in the frequency of twins and higher-order multiple births in Canada and the United States

**DOI:** 10.1186/1471-2393-12-103

**Published:** 2012-09-27

**Authors:** Deshayne B Fell, KS Joseph

**Affiliations:** 1 Better Outcomes Registry & Network (BORN) Ontario, Children’s Hospital of Eastern Ontario Research Institute, Ottawa, ON, Canada; 2 Department of Obstetrics and Gynaecology and the School of Population and Public Health, University of British Columbia and the Children’s and Women’s Hospital of British Columbia, Vancouver, BC, Canada

**Keywords:** Multiple births, Twins, Triplets

## Abstract

**Background:**

The dramatic increase in multiple births is an important public health issue, since such births have elevated risks for adverse perinatal outcomes. Our objective was to explore the most recent temporal trends in rates of multiple births in Canada and the United States.

**Methods:**

Live birth data from Canada (excluding Ontario) and the United States from 1991-2009 were used to calculate rates of twins, and triplet and higher-order multiples (triplet+). Temporal trends were assessed using tests for linear trend and absolute and relative changes in rates.

**Results:**

Twin live births in the United States increased from 23.1 in 1991 to 32.2 per 1,000 live births in 2004, remained stable between 2004 and 2007, and then increased slightly to an all-time high of 33.2 per 1,000 live births in 2009. In Canada, rates also increased from 20.0 in 1991 to 28.3 per 1,000 live births in 2004, continued to increase modestly between 2004 and 2007, and rose to a high of 31.4 per 1,000 in 2009. Rates of triplet+ live births in the United States increased dramatically from 81.4 in 1991 to 193.5 per 100,000 live births in 1998, remained stable between 1998 and 2003 and then decreased to 148.9 per 100,000 in 2007. The rate declined marginally in 2008, but then rose again in 2009 to 153.5 per 100,000. Rates of triplet+ live births were much lower in Canada, although the temporal pattern of change was similar.

**Conclusion:**

The rate of twin live births in the United States and Canada continues to increase, though more modestly than during the 1990s. Recent declines in rates of triplet+ live births in both countries have been followed by unstable trends.

## Background

The dramatic increase in multiple gestation births (i.e., twin, triplet, quadruplet and higher) over the past several decades [1-4] has been of great concern to health care providers, policy makers and researchers. Notwithstanding advances in clinical care that have improved perinatal outcomes for multi-fetal gestations [5-7], rates of preterm birth [2,6,8,9], low birth weight [2,9], fetal and infant mortality [1,6,10] and long-term developmental disability such as cerebral palsy [11,12] remain substantially higher among multiple gestation births compared with their singleton counterparts. Since the risks for these adverse outcomes tend to rise with increasing plurality [6,10,13], the rate of triplet and other higher-order multiple (triplet+) gestations has been under particularly close scrutiny.

Although some of the increase in multiple birth rates is a consequence of increased maternal age at delivery [2,3,14] (spontaneous multiple gestations arise more frequently in older women) [15], the change has been primarily attributed to an increase in the use of fertility treatments such as ovulation induction and assisted reproductive technologies (ART), i.e., *in vitro* fertilization, intracytoplasmic sperm injection, and frozen embryo transfer [16-23], which can yield iatrogenic multi-fetal gestations [24]. A high proportion of the infants born following ART-conceived pregnancies are from multiple gestations (45% in Canada in 2007 and 48% in the United States in 2006) [16,20].

In response to the high rates of multiple births and ever-improving implantation rates with ART, guidelines advocating limits on the number of embryos transferred during ART procedures emerged in Canada (in 2006 and 2010) [25,26] and the United States (first in 1998 [27] and most recently in 2009 [28]) in an attempt to reduce the incidence of iatrogenic triplet or higher-order gestations. In the mid-2000s, for the first time, there was an indication that rates of triplet and higher-order multiple births had begun to decline in the United States [29,30], but this trend seemed to abate toward the end of the decade [2]. Corresponding trends in Canada have not been reported. We undertook this descriptive study to examine trends in twin and triplet or higher-order (triplet+) live births in Canada and the United States between 1991 and 2009. Our primary objective was to describe the temporal trends in rates of twin and triplet+ live births in the United States, and contrast these with trends in Canada.

## Methods

We used vital statistics live birth information from Canada and the United States for the years 1991 to 2009. The total number of singleton, twin and triplet or higher-order (triplet+) live births in each year were obtained from Statistics Canada [31] and from a surveillance report in the United States [2]. We calculated rates of twin live births (per 1,000 live births) and triplet+ live births (per 100,000 live births) for Canada (excluding the province of Ontario) and for the United States. Data from Ontario were excluded from the calculation of overall rates for Canada due to data quality issues with respect to live birth registrations. In particular, the under-registration of live births may have affected the reporting of the number and rate of multiple live births [1,32]. A more in-depth discussion of this data quality problem can be found elsewhere [1,32]. Ontario results are presented separately.

The temporal analysis of twin and triplet+ rates was conducted separately. We first plotted the rates from the United States and examined the linear pattern to identify the time points at which the slope of the line changed. We then statistically assessed the temporal change in rates within the identified time periods using the Cochrane Armitage chi-square test for linear trend in proportions. Absolute and relative differences in rates, with 95% confidence intervals (CI), were calculated to quantify the magnitude of change between the beginning and end of each time period. This process was repeated with the Canadian data, assessing the temporal changes within the same time periods identified in the analysis of the United States data. Linear plots were generated using the observed rates for twins (per 1,000 live births). Similarly, 3-year moving averages of the observed rates for triplet+ live births (per 100,000 live births) were calculated with the first and last time points representing 2-year averages (i.e., the 1991 time point was calculated based on rates observed in 1991 and 1992, and the 2009 time point was based on rates observed in 2008 and 2009). With exception of the plots, all data preparation and analyses were conducted using SAS version 9.2 for Windows (SAS Institute Inc, Cary, NC).

A secondary analysis of the temporal trends was also carried out using Joinpoint software (version 3.4.3), which measures changing trends over time by selecting the best-fitting points (called joinpoints) at which the slope of the increase or decrease in rates changes significantly [33]. The results of the Joinpoint analysis confirmed the primary analysis; therefore only the primary analysis is presented.

## Results

**Figure 1:**
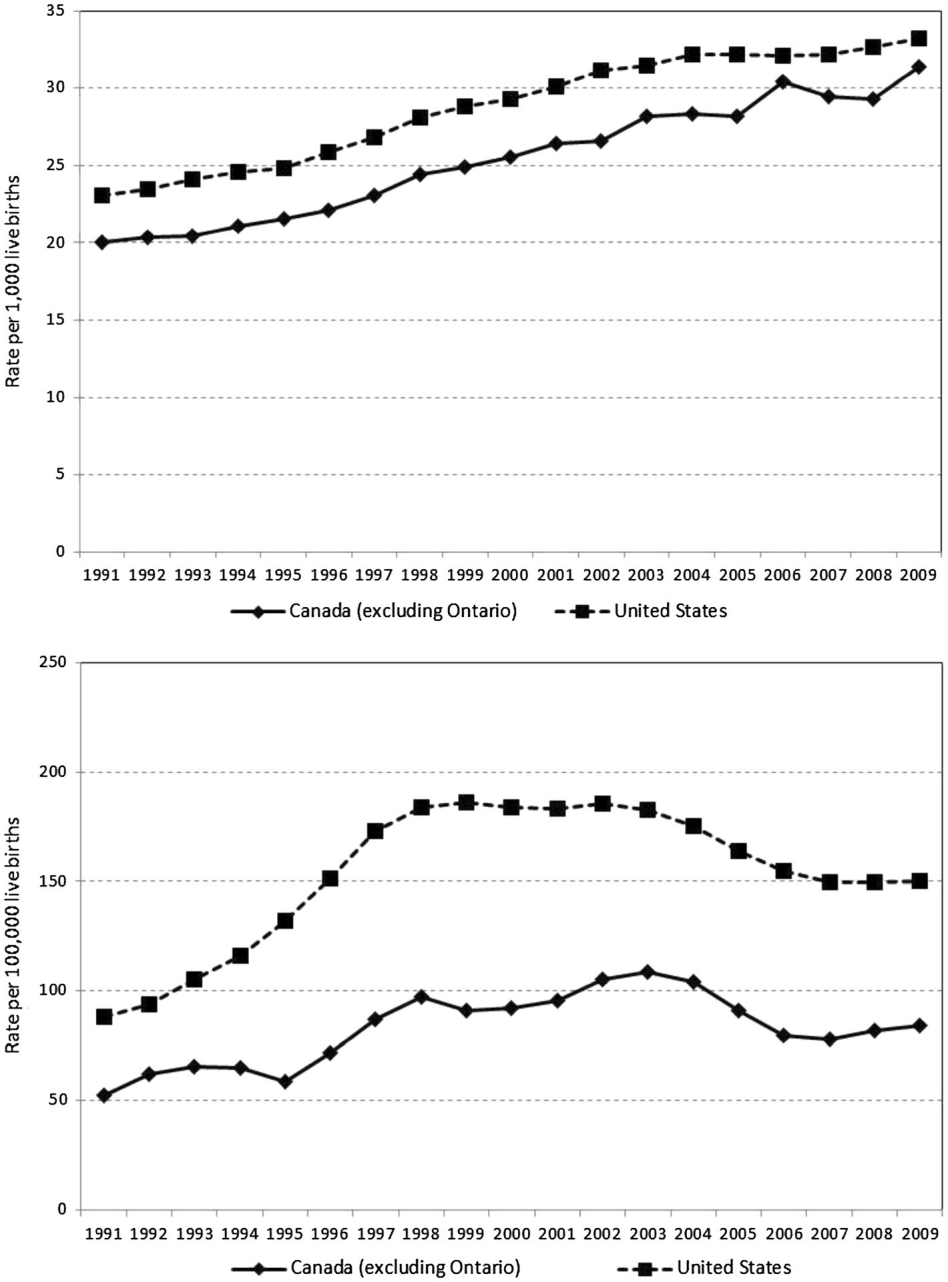
**Temporal trends in rates of twin live births (upper panel) and triplet and higher-order (triplet+) multiple live births (lower panel) in Canada (excluding Ontario), and the United States, 1991–2009.** Plots depict observed rates of twins (per 1,000 live births) and 3-year moving averages of observed rates for triplet+ (per 100,000 live births).

The rate of twin live births increased in the United States from 23.1 per 1,000 live births (95% CI: 22.9 to 23.2) in 1991, reaching a high of 32.2 per 1,000 live births (95% CI: 32.0 to 32.3) in 2004 (39% increase, P-value for trend < 0.0001; Tables 1 and 2). Between 2004 and 2007, there was little variation in the rate of twin live births (P-value for trend = 0.81; Figure 1, upper panel); however, the absolute number of such births continued to increase each year (e.g., 132,219 in 2004 to 138,961 in 2007; Table 1). Between 2007 and 2009, there was a slight decrease in the absolute number of twin live births; however, the rate increased by 1 per 1,000 to an all-time high of 33.2 per 1,000 in 2009.

**Table 1:** Number and rate* (95% confidence interval) of twins in Canada (excluding Ontario) and the United States, 1991-2009

**Year**	**Canada (excluding Ontario)**			**United States**		
	**Number of live births**	**Twin live births**		**Number of live births**	**Twin live births**	
		**Number**	**Rate (95% CI)**		**Number**	**Rate (95% CI)**
1991	250847	5027	20.0 (19.5-20.6)	4110907	94779	23.1 (22.9-23.2)
1992	247898	5053	20.4 (19.8-20.9)	4065014	95372	23.5 (23.3-23.6)
1993	240468	4920	20.5 (19.9-21.0)	4000240	96445	24.1 (24.0-24.3)
1994	238069	5013	21.1 (20.5-21.6)	3952767	97064	24.6 (24.4-24.7)
1995	231813	5005	21.6 (21.0-22.2)	3899589	96736	24.8 (24.7-25.0)
1996	226180	5008	22.1 (21.5-22.8)	3891494	100750	25.9 (25.7-26.0)
1997	215588	4975	23.1 (22.4-23.7)	3880894	104137	26.8 (26.7-27.0)
1998	209795	5133	24.5 (23.8-25.1)	3941553	110670	28.1 (27.9-28.2)
1999	206169	5140	24.9 (24.3-25.6)	3959417	114307	28.9 (28.7-29.0)
2000	200476	5117	25.5 (24.8-26.2)	4058814	118916	29.3 (29.1-29.5)
2001	202036	5337	26.4 (25.7-27.1)	4025933	121246	30.1 (29.9-30.3)
2002	200287	5324	26.6 (25.9-27.3)	4021726	125134	31.1 (30.9-31.3)
2003	204279	5753	28.2 (27.4-28.9)	4089950	128665	31.5 (31.3-31.6)
2004	204521	5798	28.3 (27.6-29.1)	4112052	132219	32.2 (32.0-32.3)
2005	208416	5869	28.2 (27.5-28.9)	4138349	133122	32.2 (32.0-32.3)
2006	219202	6665	30.4 (29.7-31.1)	4265555	137085	32.1 (32.0-32.3)
2007	229428	6770	29.5 (28.8-30.2)	4316233	138961	32.2 (32.0-32.4)
2008	237342	6953	29.3 (28.6-30.0)	4247694	138660	32.6 (32.5-32.8)
2009	240823	7564	31.4 (30.7-32.1)	4130665	137217	33.2 (33.0-33.4)

* Rates expressed per 1,000 live births.

**Table 2:** Temporal trends in rates of twin, and triplet and higher-order (triplet+) multiple live births in Canada (excluding Ontario) and the United States, 1991–2009

**Time period**	**Canada (excluding Ontario)**			**United States**		
	**Change in rate between first and last year of interval**		P-value*	**Change in rate between first and last year of interval**		P-value*
	**Absolute change in rate(95% CI)†**	**Relative change in rate(95% CI)**		**Absolute change in rate(95% CI)†**	**Relative change in rate(95% CI)**	
Twins						
1991–2004	8.3 (7.4 to 9.2)	41% (36% to 47%)	<0.0001	9.1 (8.9 to 9.3)	39% (38% to 41%)	<0.0001
2004–2007	1.2 (0.2 to 2.2)	4% (1% to 8%)	0.0005	0 (-0.2 to 0.3)	0% (-1% to 1%)	0.81
2007–2009	1.9 (0.9 to 2.9)	6% (3% to 10%)	0.0001	1.0 (0.8 to 1.3)	3% (2% to 4%)	<0.0001
Triplet+						
1991–1998	45.0 (29.0 to 61.1)	86% (50% to 132%)	<0.0001	112.1 (106.9 to 117.2)	138% (128% to 148%)	<0.0001
1998–2003	13.9 (-5.8 to 33.5)	14% (-5% to 38%)	0.04	-6.1 (-12.1 to -0.1)	-3% (-6% to 0%)	0.13
2003–2007	-33.5 (-51.9 to -15.1)	-30% (-43% to -15%)	<0.0001	-38.5 (-44.0 to -32.9)	-21% (-23% to -18%)	<0.0001
2007–2009	5.9 (-10.3 to 22.1)	6% (-12% to 32%)	0.49	4.6 (-0.7 to 9.8)	3% (0% to 7%)	0.09

* Two-sided Cochrane-Armitage test for linear trend. † Absolute change in rate per 1,000 live births for twins and per 100,000 live births for triplet+.

The rate of triplet+ live births in the United States also increased, but much more dramatically (Table 3). Between 1991 and 1998 the rate increased by 112 per 100,000 live births (P-value for trend <0.0001; Tables 2 and 3), from 81.4 per 100,000 (95% CI: 78.7 to 84.2) to 193.5 per 100,000 (95% CI: 189.2 to 197.9). Between 1998 and 2003, the rate was relatively stable (Figure 1, lower panel), and this was followed by a statistically significant decline in rates between 2003 and 2007 (absolute reduction of 38 per 100,000 live births, P-value for trend <0.0001; Tables 2 and 3). The rate declined marginally in 2008, but then rose again in 2009 to 153.5 per 100,000 (95% CI: 149.7 to 157.3). In 2007, the absolute number of triplet+ live births in the United States was its lowest value in more than a decade (6,427). Despite the small, non-significant rate increase since 2007, the absolute number of triplet+ live births in 2008 and 2009 was lower than in 2007 (i.e., 6,268 in 2008 and 6,340 in 2009).

**Table 3:** Number and rate* (95% confidence interval) of triplet and higher-order (triplet+) multiple live births in Canada (excluding Ontario) and the United States, 1991–2009

**Year**	**Canada (excluding Ontario)**			**United States**		
	**Number of live births**	**Triplet+ live births**		**Number of live births**	**Triplet+ live births**	
		**Number**	**Rate (95% CI)**		**Number**	**Rate (95% CI)**
1991	250847	131	52.2 (43.7-62.0)	4110907	3346	81.4 (78.7-84.2)
1992	247898	130	52.4 (43.8-62.3)	4065014	3883	95.5 (92.6-98.6)
1993	240468	196	81.5 (70.5-93.8)	4000240	4168	104.2 (101.1-107.4)
1994	238069	148	62.2 (52.5-73.0)	3952767	4594	116.2 (112.9-119.6)
1995	231813	118	50.9 (42.1-61.0)	3899589	4973	127.5 (124.0-131.1)
1996	226180	141	62.3 (52.5-73.5)	3891494	5939	152.6 (148.8-156.6)
1997	215588	218	101.1 (88.1-115.5)	3880894	6737	173.6 (169.5-177.8)
1998	209795	204	97.2 (84.4-111.5)	3941553	7625	193.5 (189.2-197.9)
1999	206169	194	94.1 (81.4-108.3)	3959417	7321	184.9 (180.7-189.1)
2000	200476	164	81.8 (69.8-95.3)	4058814	7325	180.5 (176.4-184.6)
2001	202036	202	100.0 (86.7-114.8)	4025933	7471	185.6 (181.4-189.8)
2002	200287	209	104.4 (90.7-119.5)	4021726	7401	184.0 (179.8-188.3)
2003	204279	227	111.1 (97.2-126.5)	4089950	7663	187.4 (183.2-191.6)
2004	204521	228	111.5 (97.5-127.0)	4112052	7275	176.9 (172.9-181.1)
2005	208416	187	89.7 (77.3-103.5)	4138349	6694	161.8 (157.9-165.7)
2006	219202	157	71.6 (60.9-83.8)	4265555	6540	153.3 (149.6-157.1)
2007	229428	178	77.6 (66.6-89.8)	4316233	6427	148.9 (145.3-152.6)
2008	237342	202	85.1 (73.8-97.6)	4247694	6268	147.6 (143.9-151.2)
2009	240823	201	83.5 (72.3-95.8)	4130665	6340	153.5 (149.7-157.3)

* Rates expressed per 100,000 live births.

The temporal pattern in rates of twin live births in Canada (excluding Ontario) closely paralleled that of the United States –– a 41% increase (P-value for trend <0.0001) was observed between 1991 and 2004 (from 20.0 per 1,000 live births to 28.3 per 1,000; Tables 1 and 2). Unlike the United States, however, the rate of twin live births continued to rise modestly, but significantly, in Canada between 2004 and 2007 (4% increase, P-value for trend 0.0005; Tables 1 and 2). The absolute number of twin live births in Canada (excluding Ontario) also continued to rise. The rate increase also persisted between 2007 to 2009, rising by about 2 per 1,000 live births from 29.5 (95% CI: 28.8-30.2) to 31.4 (95% CI: 30.7-32.1) and this was accompanied by an increase in the absolute number of twin live births in each successive year (e.g., 6,770 in 2007 to 7,564 in 2009; Table 1).

The rate of triplet+ live births in Canada (excluding Ontario) was much lower and demonstrated far more variability than the rate in the United States (Figure 1, lower panel). Nevertheless, the temporal pattern was similar –– between 1991 and 1998, the rate of triplet+ live births increased significantly (86% increase, P-value for trend <0.0001; Tables 2 and 3). From 1998 to 2003, no consistent change was observed in the rate; however, this was followed by a significant decline by about 33 per 100,000 between 2003 and 2007 (from 111.1 per 100,000 live births to 77.6 per 100,000, P-value for trend <0.0001). Similar to the United States, the rate of triplet+ live births increased non-significantly between 2007 and 2009.

The temporal patterns for twin live births in Ontario were similar to the rest of Canada (Additional file 1, upper panel). Nevertheless, rates of twin live births in Ontario were consistently higher than in the rest of Canada, approached the rates observed in the United States, and even surpassed them in 2009 (when rates in Ontario were 33.9 per 1,000 live births versus 33.2 per 1,000 in the United States). Similarly, the rate of triplet+ live births in Ontario was, on average, considerably higher than in the rest of Canada (e.g., 143.2 per 100,000 live births versus 83.5 per 100,000, respectively, in 2009). As in the rest of Canada and the United States, the rate of triplet+ live births in Ontario declined between 2003 and 2007; however, there was a statistically significant absolute increase of 35 per 100,000 triplet+ live births between 2007 and 2009 (Additional file 1, lower panel).

## Discussion

Throughout the 1990s and the early part of the subsequent decade, there was a dramatic rise in rates of multiple births in Canada and the United States. Our examination of trends over close to two decades demonstrates that the increasing frequency of twin live births has recently slowed, especially in the United States. Rates of triplet and higher-order multiple births decreased in the mid-2000s, both in Canada and the United States; however, in the latter part of the decade the declining rates leveled off and showed some modest inclination toward a further increase, though this was not statistically significant.

The recent decline in rates of higher-order multiple births is noteworthy insofar as it occurred despite increasing use of ART procedures by women seeking assistance to achieve pregnancy. In the United States, the number of ART cycles increased from 99,629 in 2000 to 146,244 in 2009 [23], and in Canada, the number of reported ART procedures increased by about 21% between 2003 and 2007 (from 10,656 to 13,482) [16,34]. One possible explanation for the opposing trends in rates of triplet+ live births and number of ART procedures is that there has been a change in clinical practice related to assisted reproduction, including primary prevention of triplet+ gestations by limiting the number of embryos transferred during ART, or by reducing triplet and higher-order gestations to twin or singleton gestations through multi-fetal pregnancy reduction [30]. Indeed, the former explanation appears likely given that in the United States, the proportion of *in vitro* fertilization procedures (using fresh eggs or embryos) in which a single embryo was transferred increased from about 6% in 2000 to about 14% in 2009, and there was a corresponding decrease in the proportion of transfers of three or more embryos (from about 69% in 2000 to 35% in 2009) [23]. The proportion of all ART births that are higher-order multiples has also decreased in the United States [29]. In Canada, the proportion of ART procedures in which three or more embryos are transferred was 31% in 2007 with little change between 2004 and 2007 [16-19].

There is tremendous variability in embryo transfer practices internationally, influenced by legislation, availability of public funding for ART, and clinical as well as social factors [35]. Reviews of international policies and practices related to ART have documented the highest rates of single embryo transfer in Sweden, Australia, New Zealand and some other Scandinavian countries, with rates in Canada and the United States among the lowest of those studied [35,36]. Further, those countries with the highest proportion of single embryo transfers also had the highest rates of singleton pregnancies following ART [36]. In the Canadian province of Quebec, a recent study reported a substantial increase in elective single embryo transfers and concomitant reduction in multi-fetal pregnancies in the first three months following the implementation in 2010 of public funding for ART and new legislation [37] mandating single embryo transfer (except under specific circumstances) [38]. Given that single embryo transfer reduces the incidence of iatrogenic multi-fetal gestations [39], the impact of clinical practice guidelines [25,26,28] and legislation [37] on embryo transfer practices and rates of multiple births following ART requires further scrutiny in Canada in the coming years.

This study is descriptive and thus cannot provide conclusive explanations for the observed temporal trends. Our source of data for the United States did not contain information on fetal deaths, and thus we restricted our analyses to live births. The exclusion of stillbirths from our calculations would have resulted in lower overall rates of multiple births and such underestimation would have been relatively greater for triplet and higher-order gestations and for earlier years of the study (given higher fetal mortality in higher-order multiple gestations and in the past [5]). Live births from Ontario were excluded from the overall Canadian rates even though about 40% of Canadian live births occur in this province [40]. However, the documented problems with under-registration of live births [1,32] have the potential to affect the accuracy of the number and rates of multiple live births.

## Conclusion

In conclusion, temporal patterns in rates of twin and triplet+ live births were similar in the United States and Canada, though triplet+ rates were much lower in Canada. Rates of twin live births have continued to increase in both countries in the 2000s, though modestly compared with the increases observed in the 1990s. The encouraging decline in rates of triplet and higher-order multiple live births that was observed in both countries in the mid-2000s waned between 2007 and 2009. While the recent decrease in triplet+ rates is important, the rates and corresponding number of infants born following a triplet gestation remains high. The fact that the decline in triplet+ rates occurred against a backdrop of increasing use of ART procedures may reflect a shift in clinical practice related to assisted reproduction (e.g., increasing use of single embryo transfer). Continued monitoring of trends in twins and higher-order multiple births and their impact on perinatal outcomes is warranted.

Addendum: The most recent data from the United States for 2010 show that the rate of twin live births remained stable at 33.1 per 1,000 live births, while the rate of triplet+ live births declined to 137.6 per 100,000 live births [41].

## Competing interests

The authors have no competing interests to declare.

## Authors’ contributions

DBF and KSJ both contributed to the conception and undertaking of the study and in the preparation of the manuscript. Both authors have reviewed and approved the final manuscript.

## Pre-publication history

The pre-publication history for this paper can be accessed here:

http://www.biomedcentral.com/1471-2393/12/103/prepub

## Supplementary Material

Additional file 1 **Figure 1.** Temporal trends in rates of twin live births (upper panel) and triplet and higher-order (triplet+) multiple live births (lower panel) in Canada (excluding Ontario), Ontario and the United States, 1991–2009. Plots depict observed rates of twins (per 1,000 live births) and 3-year moving averages of observed rates for triplet+ (per 100,000 live births). Click here for file

## References

[B1] Public Health Agency of Canada Canadian Perinatal Health Report 2008 2008 Ottawa: Minister of Health Catalogue No HP10-12/2008E

[B2] Martin JA Hamilton BE Ventura SJ Osterman MJK Kirmeyer S Mathews TJ Wilson E Division of Vital Statistics Births: final data for 2009, table 27 Natl Vital Stat Rep 2011 60 1 70 http://www.cdc.gov/nchs/data/nvsr/nvsr60/nvsr60_01.pdf. Accessed August 7, 2012 22670489

[B3] Martin JA Hamilton BE Osterman MJK Three Decades of Twin Births in the United States, 1980-2009 NCHS data brief, no 80 2012 MD: National Center for Health Statistics, Hyattsville 22617378

[B4] Martin JA Park MM Trends in twin and triplet births: 1980-97 Natl Vital Stat Rep 1999 47 1 16 11968567

[B5] Ananth CV Joseph KS Kinzler WL The influence of obstetric intervention on trends in twin stillbirths: United States, 1989-99 J Matern Fetal Neonatal Med 2004 15 380 387 10.1080/14767058410001727413 15280109

[B6] Joseph KS Marcoux S Ohlsson A Kramer MS Allen AC Liu S Wu Wen S Demissie K Sauve R Liston R for the Fetal and Infant Health Study Group of the Canadian Perinatal Surveillance System Preterm birth, stillbirth and infant mortality among triplet births in Canada, 1985-96 Paediatr Perinat Epidemiol 2002 16 141 148 10.1046/j.1365-3016.2002.00413.x 12064268

[B7] Getahun D Amre DK Ananth CV Demissie K Rhoads GG Temporal changes in rates of stillbirth, neonatal and infant mortality among triplet gestations in the United States Am J Obstet Gynecol 2006 195 1506 1511 10.1016/j.ajog.2006.01.043 16677587

[B8] Liu S Allen A Fraser W Preterm birth rate Canadian Perinatal Health Report 2008 2008 Ottawa: Public Health Agency of Canada 123 126

[B9] Blondel B Kogan MD Alexander GR Dattani N Kramer MS Macfarlane A Wen SW The impact of the increasing number of multiple births on the rates of preterm birth and low birthweight: an international study Am J Pub Health 2002 92 1323 1330 10.2105/AJPH.92.8.1323 12144992 PMC1447238

[B10] Mathews TJ MacDorman MF Infant mortality statistics from the 2007 period linked birth/infant death data set Natl Vital Stat Rep 2011 59 1 31 21957694

[B11] Rand L Eddleman KA Stone J Long-term outcomes in multiple gestations Clin Perinatol 2005 32 495 513 10.1016/j.clp.2005.03.002 15922795

[B12] Pharoah POD Risk of cerebral palsy in multiple pregnancies Clin Perinatol 2006 33 301 313 10.1016/j.clp.2006.03.017 16765726

[B13] Blickstein I How and why are triplets disadvantaged compared to twins? Best Pract Res Clin Obstet Gynaecol 2004 18 631 644 10.1016/j.bpobgyn.2004.04.014 15279822

[B14] Huang L Royle C Boscoe M Rate of live births to older mothers Canadian Perinatal Health Report 2008 2008 Ottawa: Public Health Agency of Canada 67 71

[B15] Blondel B Kaminski M Trends in the occurrence, determinants, and consequences of multiple births Semin Perinatol 2002 26 239 249 10.1053/sper.2002.34775 12211614

[B16] Gunby J Bissonnette F Librach C Cowan L IVF Directors Group of the Canadian Fertility and Andrology Society Assisted reproductive technologies (ART) in Canada: 2007 results from the Canadian ART register Fertil Steril 2011 95 542 547 e1-10. Epub 2010 Jul 24 10.1016/j.fertnstert.2010.05.057 20656287

[B17] Gunby J Bissonnette F Librach C Cowan L IVF Directors Group of the Canadian Fertility and Andrology Society Assisted reproductive technologies (ART) in Canada: 2006 results from the Canadian ART register Fertil Steril 2010 93 2189 2201 Epub 2009 May 12 10.1016/j.fertnstert.2009.03.102 19439295

[B18] Gunby J Bissonnette F Librach C Cowan L IVF Directors Group of the Canadian Fertility and Andrology Society Assisted reproductive technologies in Canada: 2005 results from the Canadian assisted reproductive technologies register Fertil Steril 2009 91 1721 1730 Epub 2008 Apr 18 10.1016/j.fertnstert.2008.02.125 18423460

[B19] Gunby J Bissonnette F Librach C Cowan L IVF Directors Group of the Canadian Fertility and Andrology Society Assisted reproductive technologies (ART) in Canada: 2004 results from the Canadian ART register Fertil Steril 2008 89 1123 1132 Epub 2007 Aug 13 10.1016/j.fertnstert.2007.05.015 17706210

[B20] Sunderam S Chang J Flowers L Kulkarni A Sentelle G Jeng G Macaluso M Centers for Disease Control and Prevention (CDC) Assisted reproductive technology surveillance – United States, 2006 MMWR Surveill Summ 2009 58 SS-5 1 25 19521336

[B21] Wright VC Chang J Jeng G Macaluso M Centers for Disease Control and Prevention (CDC) Assisted reproductive technology surveillance – United States, 2005 MMWR Surveill Summ 2008 57 1 23 18566567

[B22] Wright VC Chang J Jeng G Chen M Macaluso M Centers for Disease Control and Prevention (CDC) Assisted reproductive technology surveillance – United States, 2004 MMWR Surveill Summ 2007 56 1 22 17557073

[B23] Centers for Disease Control and Prevention, American Society for Reproductive Medicine, Society for Assisted Reproductive Technology 2009 Assisted Reproductive Technology Success Rates: National Summary and Fertility Clinic Reports 2011 Atlanta: Department of Health and Human Services http://www.cdc.gov/art/ART2009/PDF/ART_2009_Full.pdf. Accessed February 22, 2012

[B24] Blickstein I The worldwide impact of iatrogenic pregnancy Int J Gynaecol Obstet 2003 82 307 317 10.1016/S0020-7292(03)00219-4 14499977

[B25] Min JK Claman P Hughes E Society of Obstetricians and Gynecologists of Canada; Canadian Fertility and Andrology Society Guidelines for the number of embryos to transfer following in vitro fertilization. Joint SOGC–CFAS guideline No. 182 J Obstet Gynaecol Can 2006 28 799 813 17022921 10.1016/S1701-2163(16)32246-0

[B26] Min JK Hughes E Young D Elective single embryo transfer following in vitro fertilization. Joint SOGC–CFAS clinical practice guideline No. 241 J Obstet Gynaecol Can 2010 32 363 377 20500945 10.1016/S1701-2163(16)34482-6

[B27] Dickey RP The relative contribution of assisted reproductive technologies and ovulation induction to multiple births in the United States 5 years after the society for assisted reproductive technology/American society for reproductive medicine recommendation to limit the number of embryos transferred Fertil Steril 2007 88 1554 1561 10.1016/j.fertnstert.2007.01.112 17481621

[B28] American society for reproductive medicine and society for assisted reproductive technology Guidelines on number of embryos transferred Fertil Steril 2009 92 1518 1519 10.1016/j.fertnstert.2009.08.059 19836732

[B29] Martin JA Hamilton BE Sutton PD Ventura SJ Mathews TJ Kirmeyer S Osterman MJK Births: final data for 2007 Natl Vital Stat Rep 2010 58 1 85 21254725

[B30] Blickstein I Keith LG The decreased rates of triplet births: temporal trends and biologic speculations Am J Obstet Gynecol 2005 193 327 331 10.1016/j.ajog.2005.01.007 16098851

[B31] Live births database Canadian Vital Statistics System, 1991-2009 2011 Ottawa: Statistics Canada

[B32] Woodward GL Bienefeld MK Ardal S Under-reporting of live births in Ontario: 1991-1997 Can J Public Health 2003 94 463 467 14700248 10.1007/BF03405086 PMC6980132

[B33] National Cancer Institute Joinpoint Regression Program, Version 3.4.3 2010 Statistical Methodology and Applications Branch and Data Modeling Branch, Surveillance Research Program, National Cancer Institute

[B34] Gunby J Daya S IVF Directors Group of the Canadian Fertility and Andrology Society Assisted reproductive technologies (ART) in Canada: 2003 results from the Canadian ART register Fertil Steril 2007 88 550 559 Epub 2007 Feb 14 10.1016/j.fertnstert.2006.11.156 17303133

[B35] Maheshwari A Griffiths S Bhattacharya S Global variation in the uptake of single embryo transfer Hum Reprod Update 2011 17 107 120 10.1093/humupd/dmq028 20634207

[B36] Cook JL Collins J Buckett W Racowsky C Hughes E Jarvi K Assisted reproductive technology-related multiple births: Canada in an international context J Obstet Gynaecol Can 2011 33 159 167 21352636 10.1016/S1701-2163(16)34803-4

[B37] National Assembly of Quebec Bill 26: An act respecting clinical and research activities related to assisted procreation 2009 http://www2.publicationsduquebec.gouv.qc.ca/dynamicSearch/telecharge.php?type = 5&file = 2009C30A. PDF. Accessed February 22, 2012

[B38] Bissonnette F Phillips SJ Gunby J Holzer H Mahutte N St-Michel P Kadoch U Working to eliminate multiple pregnancies: a success story in Québec Reprod Biomed Online 2011 23 500 504 10.1016/j.rbmo.2011.05.020 21840757

[B39] Karlstrom PO Bergh C Reducing the number of embryos transferred in Sweden-impact on delivery and multiple birth rates Hum Reprod 2007 22 2202 2207 10.1093/humrep/dem120 17562674

[B40] Statistics Canada Births 2009. Table 1 Catalogue no. 84F0210X. http://www.statcan.gc.ca/pub/84f0210x/84f0210x2009000-eng.pdf. Accessed August 31, 2012

[B41] Martin JA Hamilton BE Ventura SJ Osterman MJK Wilson E Mathews TJ Division of Vital Statistics Births: final data for 2010, table 27 Natl Vital Stat Rep 2012 61 1 100 http://www.cdc.gov/nchs/data/nvsr/nvsr61/nvsr61_01.pdf. Accessed August 31, 2012 24974589

