# Supplementary material for: Temporal trends in the frequency of twins and higher-order multiple births in Canada and the United States
Source: BMC Pregnancy Childbirth. 2012 Sep 27;12:103. doi: 10.1186/1471-2393-12-103 (PMC3533860; doi:10.1186/1471-2393-12-103)

**APPENDIX**

**Figure title**

Figure 1. Temporal trends in rates of twin live births (upper panel) and triplet and higher-order (triplet+) multiple live births (lower panel) in Canada (excluding Ontario), Ontario and the United States, 1991–2009.

**Figure legend**

Plots depict observed rates of twins (per 1,000 live births) and 3-year moving averages of observed rates for triplet+ (per 100,000 live births).


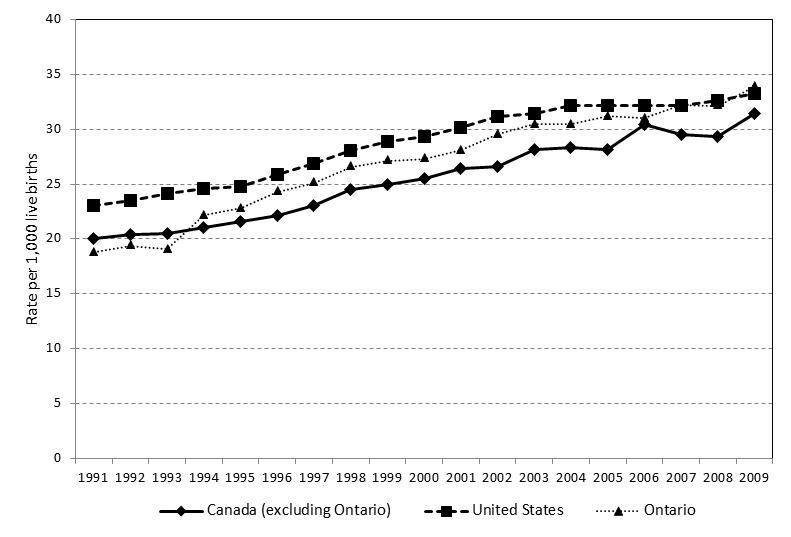


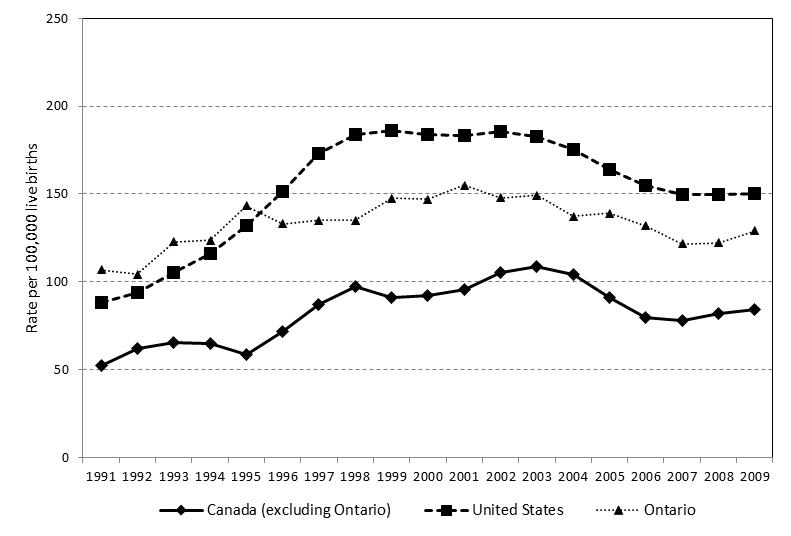

Supplement: Additional file 1 — Figure 1. Temporal trends in rates of twin live births (upper panel) and triplet and higher-order (triplet+) multiple live births (lower panel) in Canada (excluding Ontario), Ontario and the United States, 1991–2009. Plots depict observed rates of twins (per 1,000 live births) and 3-year moving averages of observed rates for triplet+ (per 100,000 live births). [file 1471-2393-12-103-S1.doc]
